# Development and Characterization of 3D-Printed PLA/Exfoliated Graphite Composites for Enhanced Electrochemical Performance in Energy Storage Applications

**DOI:** 10.3390/polym16223131

**Published:** 2024-11-09

**Authors:** Ananias Lima dos Santos, Francisco Cezar Ramos de Souza, João Carlos Martins da Costa, Daniel Araújo Gonçalves, Raimundo Ribeiro Passos, Leandro Aparecido Pocrifka

**Affiliations:** GEMATA—LEEN, Department of Chemistry, University Federal of Amazonas, Manaus 69067-005, AM, Brazil; ananias_sbp@hotmail.com (A.L.d.S.); cezrmos@gmail.com (F.C.R.d.S.); jcarlosmartins.89@gmail.com (J.C.M.d.C.); daniel.araujogoncalves@gmail.com (D.A.G.); rrpassos@ufam.edu.br (R.R.P.)

**Keywords:** additive manufacturing, composite materials, polylactic acid (PLA), exfoliated graphite (EG), cyclic voltammetry, electrochemical impedance spectroscopy

## Abstract

This research introduces a new way to create a composite material (PLA/EG) for 3D printing. It combines polylactic acid (PLA) with exfoliated graphite (EG) using a physical mixing method, followed by direct mixing in a single-screw extruder. Structural and vibrational analyses using X-ray diffraction and Fourier transform infrared spectroscopy confirmed the PLA/EG’s formation (composite). The analysis also suggests physical adsorption as the primary interaction between the two materials. The exfoliated graphite acts as a barrier (thermal behavior), reducing heat transfer via TG. Electrochemical measurements reveal redox activity (cyclic voltammetry) with a specific capacitance of ~ 6 F g^−1^, low solution resistance, and negligible charge transfer resistance, indicating ion movement through a Warburg diffusion process. Additionally, in terms of complex behavior (electrochemical impedance spectroscopy), the PLA/EG’s actual capacitance C’(ω) displayed a value greater than 1000 μF cm^−2^, highlighting the composite’s effectiveness in storing charge. These results demonstrate that PLA/EG composites hold significant promise as electrodes in electronic devices. The methodology used in this study not only provides a practical way to create functional composites but also opens doors for new applications in electronics and energy storage.

## 1. Introduction

The quest for more efficient and sustainable materials is driving research across various scientific fields, particularly in energy storage and conversion technologies [1,2]. With the growing global demand for portable electronic devices and clean energy solutions, the development of novel electrode materials that offer superior performance, cost-efficiency, and long-term stability has become crucial [3]. In this context, additive manufacturing (AM), commonly known as 3D printing, has emerged as a powerful tool for creating functional materials with complex geometries and tailored properties [4,5].

Polylactic acid (PLA), a biodegradable thermoplastic, is widely used in AM due to its ease of processing, biocompatibility, and low environmental impact [6,7]. However, its inherent limitations in mechanical and electrical performance restrict its use in advanced applications, such as energy storage [8,9]. To overcome these limitations, recent research has focused on developing PLA-based composites by incorporating conductive fillers, particularly carbon-based materials, to enhance the electrical and mechanical properties of the resulting structures [9,10].

Among the various carbon-based materials, graphite stands out due to its high electrical conductivity, thermal stability, and cost-effectiveness [11,12,13,14]. By exfoliating graphite to increase its surface area and incorporating it into PLA [15], it is possible to create hybrid composites with improved electrochemical properties, making them suitable for applications in supercapacitors, batteries, and sensors [16,17,18].

Among the studies on this topic, González-Lopez et al. (2024) [14] used PLA and mixed it with graphite via dissolution in dichloromethane, being extruded in a monothread, thus forming a filament for PLA/graphite 3D printing, in order to observe whether or not there were changes in the new material formed. According to the characterization methods adopted, including Raman spectroscopy techniques, Fourier transform infrared (FTIR), and thermogravimetric analysis (TGA), it was observed that there were no significant changes in the material, while the characterization via Raman spectroscopy showed that the formed materials transformed into composites.

When performing electrical analyses via cyclic voltammetry (CV) using 0.5 mol L^−1^ sodium hydroxide (NaOH) as an electrolyte, which was previously activated in the same solution for 200 s in a potential window of −1 to 1.4 V, after the activation step, the CV tests occurred in the potential range of −1 to 1 V, and it was observed that the formed material reached a current density of 200 μA. Under the conditions employed, the authors concluded that the composite had attributes suitable for electrochemical sensors.

Faria et al. (2023) [19] studied the formation of a composite of PLA and graphite, with PLA dissolved in a solution composed of chloroform/acetone, to which graphite particles were added and, via extrusion, the filament for 3D printing was formed. The formed samples were characterized via thermogravimetric analysis and Raman spectroscopy and scanning electron microscopy (SEM), which confirmed that the materials obtained were composites of PLA and carbonaceous material.

To verify the electrical behavior, the material was characterized via cyclic voltammetry (CV) and the electrolyte (probe) adopted was a solution of 1 mmol.L^−1^ potassium ferrocyanide (Fe(CN)_6_) and 0.1 mol L^−1^ potassium chloride (KCl). Through this analysis, it was observed that the material formed reached a current of approximately 20 μA, while according to electrochemical impedance spectroscopy (EIS), it displayed an ohmic resistance of 24 KΩ. The results obtained in this study indicate that the material and methodology can be used for the quality control of pharmaceutical products and the protection of the environment.

Cardoso et al. (2020) [20] adopted PLA as an object of study in order to form composites with PLA/carbon black, in which PLA was dissolved in dopamine and carbon black was later added in different proportions, and then a filament for 3D printing was extruded. The formed samples were characterized via Raman spectroscopy and SEM, and the results confirmed that the formation of PLA/carbon black composites occurred.

In the electrochemical analysis, the electrolyte (probe) used was a solution of 1 mol L^−1^ Fe(CN)_6_, 0.1 mol L^−1^ potassium chloride (KCl), and 0.1 mol L^−1^ sulfuric acid (H_2_SO_4_). Via cyclic voltammetry, it was observed that the material obtained a current of approximately 18 μA; it was also tested in a solution of 100 μL of trinitrotoluene (TNT), and the material formed in this research had potential as a sensor for the TNT compound.

With these studies in mind, this work aimed to study the formation of a PLA/exfoliated graphite (EG) composite; the graphite was exfoliated via high-energy grinding, and the incorporation of the EG also took place via high-energy grinding, while the composite was later extruded and 3D printed by means of the FDM method.

Once the materials were formed, they were analyzed via X-ray diffraction (XRD), Fourier transform infrared spectroscopy (FTIR), and thermogravimetric analysis (TGA). Their electronic properties were observed via cyclic voltammetry (CV) and electrochemical impedance spectroscopy (EIS). Based on the EIS behavior, a study was carried out in terms of complex capacitance behavior, aiming at developing a more detailed understanding of the capacitive processes and the capacitance values of PLA/EG as an electrode in energy storage devices.

## 2. Materials and Methods

### 2.1. Graphite Exfoliation

Graphite (National, 99.5%) exfoliation was performed using a high-energy ball mill (Spex 8000, SPEX^®^ Sample Prep, Metuchen, NJ, USA). A stainless-steel milling jar with four (4) stainless-steel balls was filled with 3 g of the precursor material (graphite), and milling was conducted at a rotational speed of 1024 revolutions per minute (RPM) for 12 h. This method enabled efficient mechanical exfoliation, leading to the formation of exfoliated graphite (EG).

### 2.2. Preparation and Production of PLA/EG Composite Filament via 3D Printing

This study employed a novel physical mixing method using a ball mill to prepare the PLA/exfoliated graphite (PLA/EG) composites. This innovative approach was selected for its ability to ensure a homogeneous distribution of the exfoliated graphite within the PLA (NaturalWorks, Plymouth, MN, USA) matrix while avoiding the complexities and environmental concerns associated with traditional chemical synthesis routes.

The PLA/EG composite filament was prepared using a physical mixing approach. The blending was performed in a high-energy ball mill (Spex 8000) operating at 1024 rpm for 2 h. The mixture consisted of 19.00 g of PLA pellets and 1.00 g of exfoliated graphite, resulting in a total of 20.00 g of the composite material. Following the homogenization step, the composite mixture was extruded using a Filmaq 3D extruder at a temperature of 200 °C, producing a filament with a consistent diameter Ø of 1.75 mm.

**Scheme 1:**
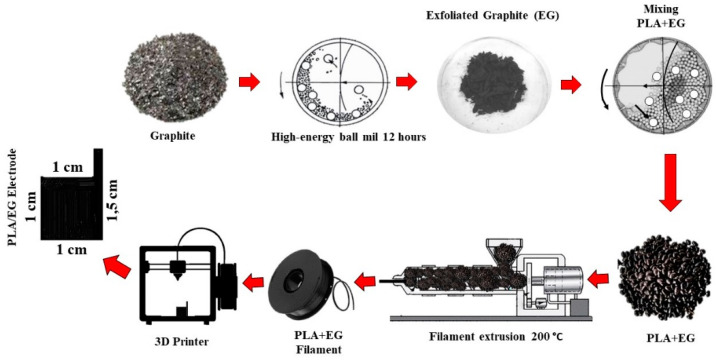
Preparation and production of PLA/EG filaments via 3D printing.

The PLA/EG filament was then used in the manufacturing of test samples via fused deposition modeling (FDM) with a 3D printer (Ender-3S1-Pro). The printing parameters included a nozzle temperature of 200 °C, a bed temperature of 70 °C, and a printing speed of 30.00 mm/s. The 0.10 mm layer height balanced resolution and time, with 100% infill and orientation along the x-axis. The 90° raster angle optimized interlayer contact and surface smoothness. The fabricated specimens were printed with a surface area of 1.00 cm^2^ (as shown in Scheme 1). The operation conditions were based on the work by González-Lopez et al. (2024) [14].

### 2.3. Characterization Techniques

X-ray diffraction (XRD) analysis was performed using a XRD-7000 diffractometer equipped (Shimadzu, Kyoto, Japan) with a Cu anode (Kα radiation, λ = 0.154 nm). The diffraction patterns were recorded in the 2θ range of 10° to 60° to identify the crystalline structures of the composites. Fourier transform infrared (FTIR) spectroscopy was conducted in the spectral range of 3600 to 500 cm^−1^ using a IR Tracer-100 spectrophotometer (Shimadzu, Kyoto, Japan) employing attenuated total reflectance (ATR) spectroscopy to analyze the vibrational structures of the PLA/EG. This analysis was used to evaluate the functional groups and vibrational structures present in the PLA/EG composite. Thermal stability and decomposition behaviors were assessed using a DTG-60 (Shimadzu, Kyoto, Japan) thermal analyzer. The analysis was performed from room temperature up to 800 °C, with a heating rate of 10 °C min^−1^ under a continuous nitrogen (N_2_) flow of 50.00 mL min^−1^.

Electrochemical properties were evaluated using an potentiostat/galvanostat PGSTAT302N (AutoLab Metrohm, Utrecht, The Netherlands,) in a three-electrode electrochemical cell configuration. The working electrode was fabricated from the PLA/EG composite, while a platinum wire and an Ag/AgCl electrode with saturated potassium chloride (KCl) (Synth) served as the counter and reference electrodes, respectively. The electrolyte for electrochemical activation was a 1.00 mol L^−1^ aqueous solution of potassium hydroxide (KOH) (Synth). The activation of the PLA/EG electrode was carried out through cyclic voltammetry (CV) at a scan rate of 200 mV.s^−1^ for 200 cycles in the potential range of −0.60 to 1.20 V. Subsequent electrochemical measurements, including CV and electrochemical impedance spectroscopy (EIS), were conducted using a Na_2_SO_4_ solution (1.00 mol L^−1^) mixed with 5.00 mmol L^−1^ of K_3_[Fe(CN)_6_] (Synth). CV measurements were performed within the potential window of −0.4 to 0.40 V at various scan rates (100, 50, 25, 10, 5, and 1 mV s^−1^). EIS was conducted over a frequency range from 10,000 to 0.01 Hz, using a sinusoidal voltage perturbation of 50 mV at an open-circuit potential (OCP) after a stabilization period of 600 s. These techniques provided insights into the electrochemical performance and interfacial properties of the PLA/EG composite.

## 3. Results and Discussion

The structural, thermal, and electrochemical properties of the PLA/EG composites were evaluated to understand the impact of exfoliated graphite’s (EG) incorporation into the polymer matrix. The aim was to assess the behavior of the EG synthesis route and its influence on the overall properties of the PLA-based material.

### 3.1. Structural Characterization

**Figure 1:**
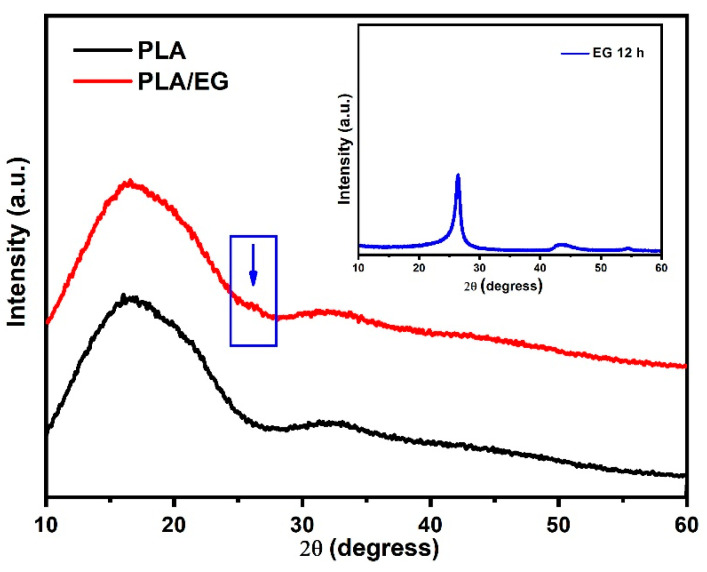
XRD standards of PLA, PLA/EG, and exfoliated graphite (EG).

The X-ray diffraction (XRD) patterns of PLA, PLA/EG, and exfoliated graphite are presented in Figure 1. The diffraction peak at 2θ = 16.4°, observed in both PLA and PLA/EG, corresponds to the (200)/(110) planes, indicating the orthorhombic crystalline structure of the PLA matrix. In the case of exfoliated graphite, a characteristic peak is observed at 2θ = 26.35°, which is consistent with the reported results for carbonaceous materials [21,22,23,24,25]. This peak is barely perceptible in the PLA/EG composite, appearing as a small bulge, suggesting that the exfoliated graphite is well dispersed within the polymer matrix. Such observations are consistent with other studies [25,26], indicating the effective incorporation of EG into the PLA structure.

**Figure 2:**
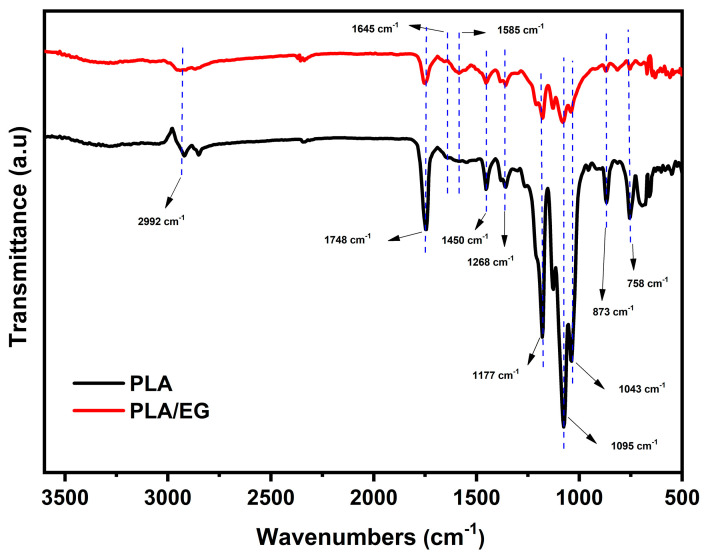
Bands observed in the FTIR spectra of PLA and PLA/EG.

Figure 2 displays the Fourier transform infrared (FTIR) spectra of PLA and PLA/EG. The typical vibrational bands of PLA can be observed, including bending vibrations at 873 cm^−1^ and 758 cm^−1^, corresponding to its amorphous and crystalline phases, respectively. The bands at 1095 cm^−1^ and 1043 cm^−1^ are attributed to the symmetric and asymmetric stretching of C–O–C and CH_3_ groups, respectively. Additionally, the band at 1748 cm^−1^ is related to C=O stretching in the ester group, while the band at 2922 cm^−1^ corresponds to CH stretching.

When EG was incorporated into the PLA matrix, significant reductions in the intensities of the bands at 1095 cm^−1^ (C–O–C stretching) and 1043 cm^−1^ (CH_3_ stretching) were observed. This attenuation effect is linked to the presence of EG, which interferes with the transmittance properties of the material [23,24,25]. Przekop et al. [26] explain that this phenomenon is related to the presence of carbonaceous material that causes the loss of transmittance of the material.

The addition of EG (reinforcement) to the PLA (matrix) is characterized by the bands at 1645 and 1585 cm^−1^ that correspond to the C=C and C–C elongations of the carbon structure of the exfoliated graphite, as reported by Przekop et al. [26]. The new bands found at 1645 and 1585 cm^−1^ in PLA/EG are attributed to the stretching vibration of the carbonaceous material, thus confirming that in the incorporation of EG into the PLA matrix, the interactions occur via adsorption between the matrix and the reinforcements in a physical mode, and reinforcement contacts occur between the superficial methyl groups and the (–C=C) group.

### 3.2. Thermal Analysis

**Figure 3:**
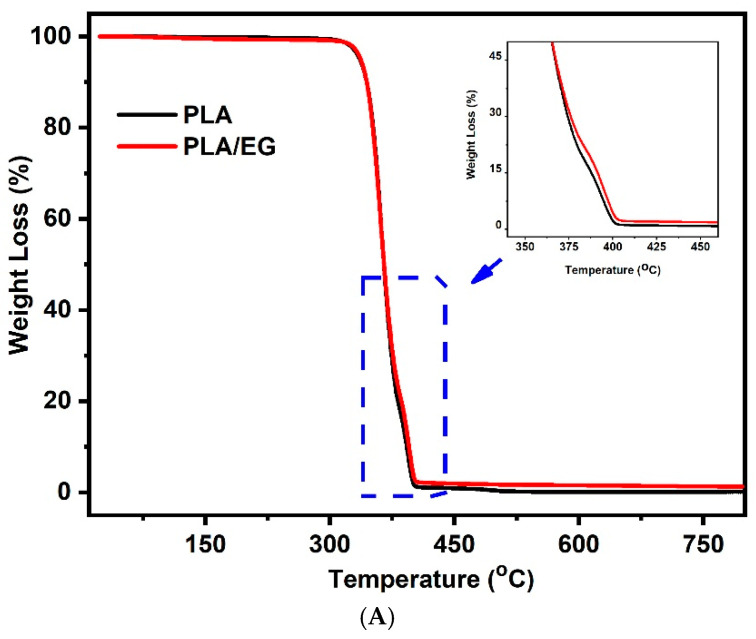

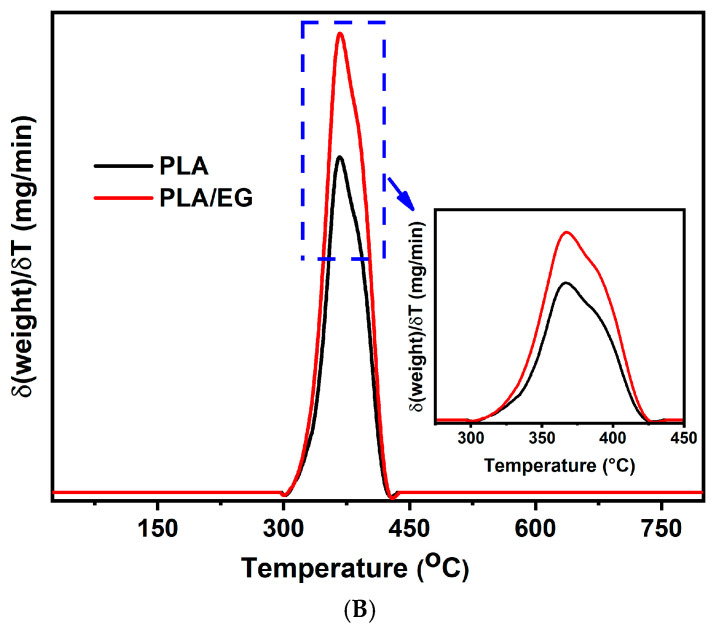
(**A**) Thermal behavior according to thermogravimetric analysis and (**B**) thermal behavior according to the thermogravimetric derivative.

Thermogravimetric analysis (TGA) curves are shown in Figure 3A for both PLA and PLA/EG. The initial degradation temperature of both materials was approximately 255 °C, and by 340 °C, the mass loss was over 70%. The final degradation process concluded around 426 °C, in agreement with studies by Abdullah et al. [27], Guo et al. [28], and Jalali et al. [29]. Regarding the initial degradation temperature and the maximum temperature at material loss (PLA/EG), the addition of exfoliated graphite to the polymer leads to a mild improvement in the thermal behavior of the composite (inset Figure 3A) when compared to PLA. Przekop et al. [26] and Guo et al. [28] also observed such phenomena in their studies and described that the addition of carbonaceous material (reinforcement) can lead to a delay in the thermodegradation effect for the polymer (matrix), which is attributed to the protective effect provided by the reinforcement.

According to differential thermogravimetry (DTG) (Figure 3B), the PLA+EG material displayed decomposition at a higher temperature, and the PLA displayed similar behavior at the same temperatures. Guo et al. [28] reported that there is an indication that the addition of EG causes a substantial thermal increase in PLA, at least in the early stages of decomposition. Silva et al. [30] reported that this improvement is mainly attributed to the good matrix/reinforcement interaction; the carbon itself has a good thermal conductivity, and due to its barrier effect, the addition of EG appears to have little effect on the temperature at which the maximum rate of decomposition occurs.

### 3.3. Electrochemical Performance

**Figure 4:**
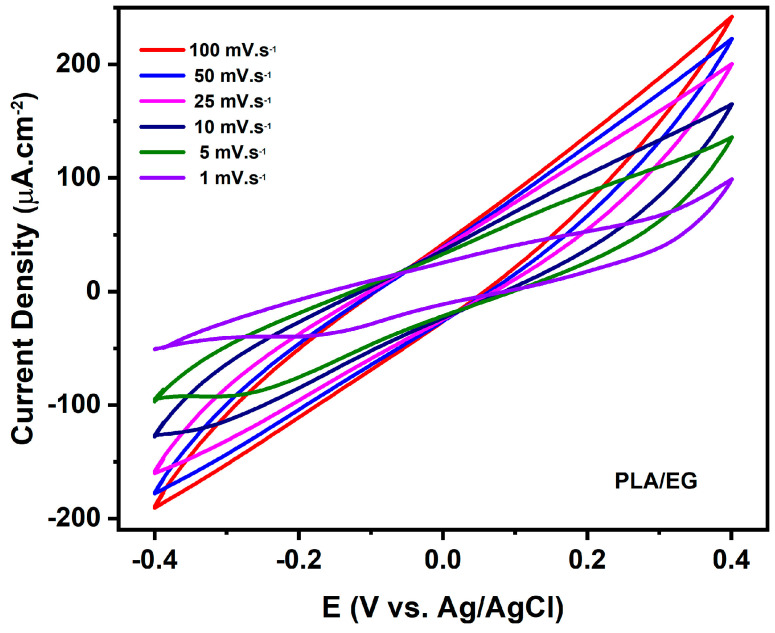
Electrochemical behavior of PLA/EG according to cyclic voltammetry.

The electrochemical behavior of PLA/EG was evaluated using cyclic voltammetry (CV) and electrochemical impedance spectroscopy (EIS). Figure 4 shows the initial activation of the PLA/EG electrode in a 1.00 mol L^−1^ KOH solution for 200 cycles at a scan rate of 200 mV s^−1^. After activation, CV analyses were performed in a Na_2_SO_4_ solution (1.00 mol L^−1^) containing 5.00 mmol L^−1^ K_3_Fe(CN)_6_. At 100 mV.s^−1^, the current density reached 240.00 μA, while at lower scan rates (1.00 mV.s^−1^), the density decreased to 10.00 μA. This behavior indicates a dependence of charge accumulation on the scan rate, which is governed by mass transport and diffusion channels within the composite [31,32].

The specific capacitance of the PLA/EG composite was determined using Equation (1), as described by Mondal et al. [32], and yielded a value of 5.97 F g^−1^. In this equation, the area is defined by the integration of the current (I) versus potential (V) loop, where C represents the specific capacitance, I is the current density, m is the mass of the electrode, v is the scan rate, and (Vf-Vi) is the potential window used in the analysis.

(1) $$C=\int \frac{I\left(V\right)dv}{mv(Vf-Vi)}$$

**Figure 5:**
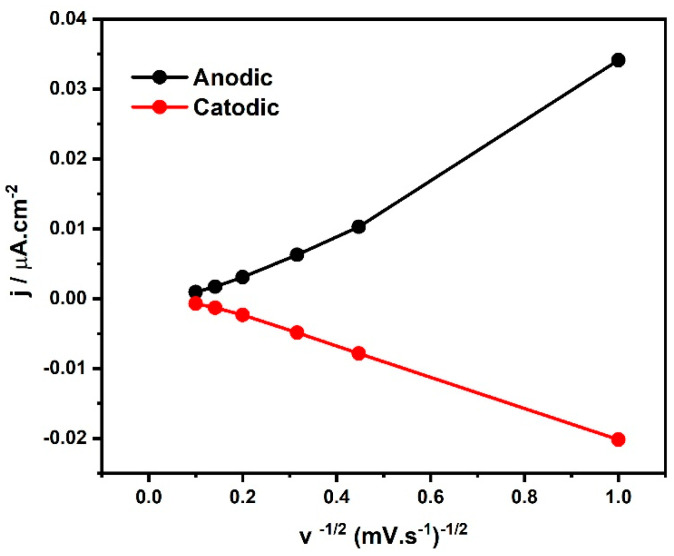
Plots of peak currents vs. the square root of scan rate, with scan rates ranging from 1 to 100 mV s^−1^.

The slopes (peak current and v, which is the scan rate) of the composite (PLA/EG) determine the diffusion rates with surface redox reactions occurring in charge storage processes, according to Silva et al. [33]. Therefore, the rates of anodic/cathodic reactions on the surface of the PLA/EG composite confirm that the Faradic reactions occur on the electrode surface (Figure 5). It is observed that with the decrease in the scan rate, there is more time for the ion diffusion through the electrode, and the calculated specific capacitance at 1 mV.s^−1^ was 5.97 F.g^−1^. As the scan rate increases, the diffusion of ions becomes limited, resulting in a lower diffusion capacity. This behavior is described in the study by Araújo et al. [34].

This result indicates that the activation process significantly enhances the interaction between the active sites of the composite and the electrolyte, thereby promoting efficient ion transport and improving the overall electrochemical performance of the material [31,35].

**Figure 6:**
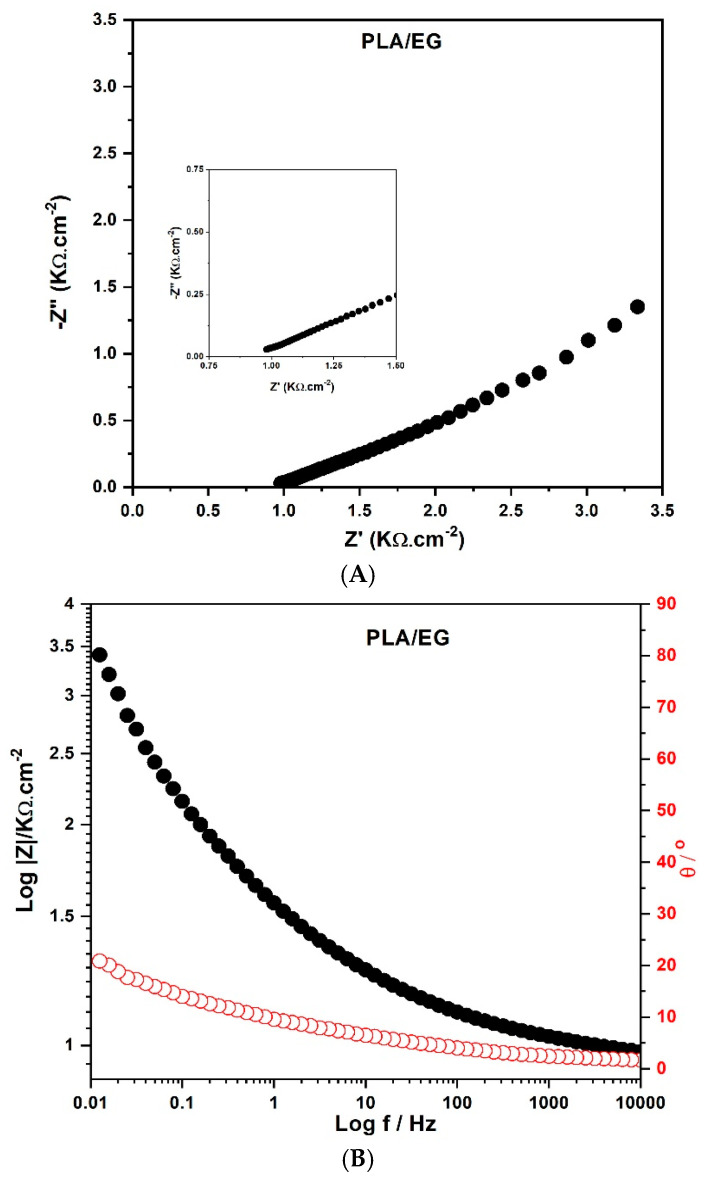

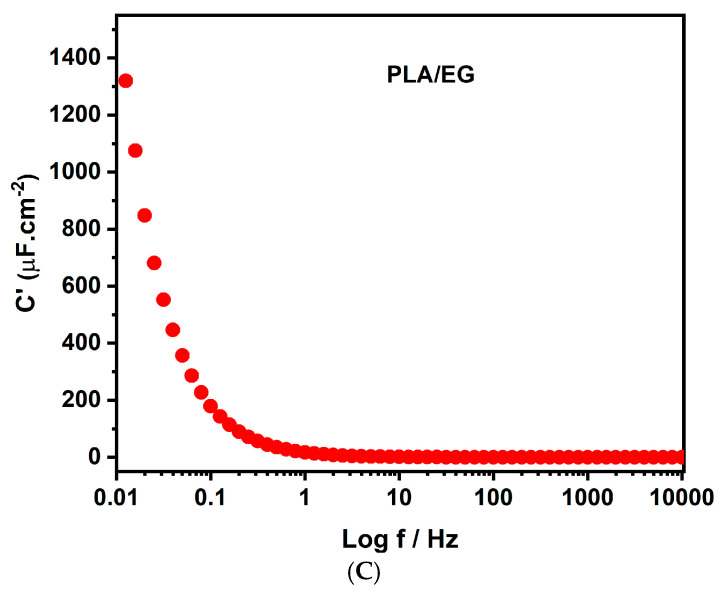
(**A**) Electrochemical behavior according to EIS via Nyquist; (**B**) electrochemical behavior according to EIS via Bode; and (**C**) real part of the complex capacitance C’(ω) of PLA/EG.

The Nyquist plots (Figure 6A) [36] show the features of the PLA/EG composite material exhibited in high-frequency ranges, where the solution resistance (Rs) was found to be 0.980 KΩ, with no charge transfer resistance (R_ct_) [37]. The lack of R_ct_ suggests that ion transport primarily relies on diffusion rather than charge transfer methods. Upon examination, the phase angle analysis (Figure 6B) reinforces this finding by revealing a phase angle of 25°. This indicates that ion transport occurs through Warburg diffusion [35,36,37].

To better understand how capacitance changes with variations in frequency and alternating currents (AC), a thorough analysis of capacitance properties was conducted, as shown in Figure 6C. By applying Equations (2)–(4) from [38,39,40], the real (C’) and imaginary (C”) capacitances were meticulously computed to delve into the distinct contributions of ions and diffusion processes within the composite material.

(2) $$C\left(\omega \right)=C”\;(\omega )-jC”(\omega )$$

(3) $$C^{\prime }\left(\omega \right)=\frac{-Z”(\omega )}{{\omega |Z\left(\omega \right)|}^{2}}$$

(4) $$C^{”}\left(\omega \right)=\frac{-Z^{\prime }(\omega )}{{\omega |Z\left(\omega \right)|}^{2}}$$

C’(ω) represents the real part of the complex capacitance, and C” (ω) the imaginary part of the complex capacitance, with C (ω) being the overall capacitance. Z’(ω) and Z”(ω) represent the real and imaginary components of the complex impedance, and ω is the angular frequency defined as ω = 2*πf*.

The findings revealed an increase in the capacitance values (C’) from an initial measurement of 5.75 μF.cm^−2^ at a frequency of 1.00 Hz to a significantly higher value of 1440 μF.cm^−2^ when the frequency was decreased to 1.00 mHz. The substantial increase in capacitance at lower frequencies indicates an improved ion mobility and better charge retention potential in the PLA/EG composite material, making it a promising candidate for energy storage applications.

The complex studies conducted in this work provided valuable insights into the ionic and diffusional contributions of the PLA/EG composite within the electrochemical system. These results were consistent with those obtained through Nyquist and Bode plots (Figure 6A,B), confirming the electrochemical responsiveness of the composite. Such behavior makes the PLA/EG composite a promising candidate for applications as electrodes in electrical and electronic devices. Additionally, the findings suggest that the employed methodology is both cost-effective and practical.

In contrast to many studies in the literature that utilize chemical synthesis routes to develop PLA-based composites, this work employed a physical mixing approach using a ball mill for the production of PLA/exfoliated graphite (PLA/EG) composites. This method not only simplifies the synthesis process but also reduces the reliance on potentially hazardous chemicals and solvents, making it a more environmentally friendly alternative. The use of a ball mill allows for the mixing of exfoliated graphite (reinforcing) with PLA (matrix), resulting in a new material without the need for complex chemical processes.

The methodology used in this study was also the subject of the work by Cavalieri et al. [41], in which it was observed that the formation of the polypropylene/polyethylene composite involved physical interactions increasing adhesion at the interfaces between the two matrix/reinforcement phases. Vertuccio et al. [42], in their work, used high-energy milling to promote the compatibility between PLC, starch, and montmorillonite.

In Table 1, a comparison is made with other works reported regarding the synthesis of PLA-based composites and their applications. The results from this work show promising potential for applications in energy storage devices. The composite exhibited electrochemical properties comparable to those reported in the literature, such as the study by González-Lopez et al. (2024) [14], who synthesized a similar PLA/carbonaceous composite using homogenization with organic solvents. This suggests that the approach used here offers a simpler and more sustainable alternative for developing functional composites with enhanced electrochemical performance.

## 4. Conclusions

This study demonstrated the feasibility of producing a novel PLA composite with exfoliated graphite (PLA/EG) using an easy and cost-effective high-energy milling process, followed by direct mixing in a single-screw extruder for 3D printing applications. The structural and vibrational analyses, confirmed by X-ray diffraction and Fourier transform infrared spectroscopy, revealed that the interaction between the polymer matrix and the exfoliated graphite occurs primarily through physical adsorption. This indicates a successful integration of EG into the PLA matrix, enhancing its properties without the complexities associated with traditional chemical methods.

The thermal analysis highlighted a mild improvement in the thermal behavior of PLA with the addition of EG, particularly in the early stages of decomposition. This enhancement is attributed to the interfacial interactions between the polymer and the carbon reinforcement, which functions as an effective thermal barrier.

Electrochemical characterization revealed that the PLA/EG composite exhibits promising capacitive behavior, with a specific capacitance of 5.97 F g^−1^ at a scan rate of 1.00 mV s^−1^ according to cyclic voltammetry, underscoring the contribution of the carbonaceous material to the redox activity. Electrochemical impedance spectroscopy demonstrated low solution resistance (Rs < 1 KΩ) and no charge transfer resistance (Rct), indicative of efficient ion transfer through Warburg diffusion mechanisms. In complex capacitance, the real capacitance reached 1440 μF cm^−2^, further emphasizing the composite’s effectiveness in energy storage applications; however, dissipation of the C” (ω) energy of the material was not observed, due to the type of ion transfer that the material experiences between the electrode and electrolyte.

Overall, the findings of this research underscore the potential of PLA/EG composites as advanced materials for use as electrodes in electronic devices, combining the sustainability of additive manufacturing with enhanced electrochemical performance. This methodology not only represents a practical approach to the fabrication of functional composites but also paves the way for future applications in the fields of electronics and energy storage. Further studies are encouraged to explore the scalability of this method and the long-term performance of PLA/EG composites in real-world applications.

## Figures and Tables

**Table 1:** Comparison of methodology and electrochemical behavior of the proposed 3D-printed PLA/carbonaceous material with other studies reported in the literature.

Papers Using 3D Printing	Percentage of Filler in the Composite	Methods for Obtaining Material	Applications	Electrochemical Behavior	References
PLA/Carbon Black	5%	Chemical homogenization	Electrochemical sensor	CV	Cardoso et al. [20]
PLA/Carbon Black	5%	Chemical homogenization	Electrochemical sensor	CV	Rocha et al. [43]
PLA/Carbon Black	5%	Chemical homogenization	Electrochemical sensor	CV	João et al. [44]
PLA/Graphite	40%	Chemical homogenization	Electrochemical sensor	CV and EIS	Faria et al. [19]
PLA/Graphite	30%	Chemical homogenization	Electrode and electrochemical sensor	CV	González-Lopez et al. [14]
PLA/Exfoliated Graphite	5%	Physical mixture	Electrode for electrochemical devices	CV and EIS	This work

Cyclic voltammetry (CV) and electrochemical impedance spectroscopy (EIS).

## Data Availability

The original contributions presented in the study are included in the article, further inquiries can be directed to the corresponding author.
